# Understanding PFAPA Syndrome in Palestine: A Retrospective Cohort Analysis of Epidemiological and Clinical Data

**DOI:** 10.31138/mjr.110824.qcr

**Published:** 2025-07-17

**Authors:** Fawzy M. Abunejma, Rose Wazwaz, Romaisa Qawasma, Raghad Abu Dabaat, Oadi N. Shrateh

**Affiliations:** 1 Faculty of Medicine, Hebron University, Palestine;; 2 Department of Paediatric Rheumatology, Palestinian Red Crescent Society Hospitals, Hebron, Palestine;; 3 Department of Paediatric Rheumatology, Al Ahli Hospital, Hebron, Palestine;; 4 Faculty of Medicine, Al-Quds University, Jerusalem, Palestine

**Keywords:** PFAPA, periodic fever, autoinflammatory syndrome, Palestine

## Abstract

**Background:**

Despite Periodic Fever, Aphthous Stomatitis, Pharyngitis, and Adenopathy being widely acknowledged as a clinical entity and considered one of the most prevalent autoinflammatory diseases, there remains controversy surrounding its diagnostic criteria. Additionally, the epidemiology of the disease is largely unknown in Palestine. Therefore, the goal of this study is to enhance the understanding of PFAPA syndrome in Palestinian paediatric patients.

**Methods:**

We carried out a retrospective cohort study that included 57 patients diagnosed with PFAPA at hospitals in Hebron, Palestine, specifically at Al-Ahli and the Palestinian Red Crescent Society (PRCS).

**Results:**

The study revealed that PFAPA patients predominantly experienced fever (93.0%) and pharyngotonsillitis (100.0%), with significant associations noted between PFAPA and the presence of pharyngotonsillitis (p=0.006), adenitis (p=0.001), and periodicity. However, no significant associations were found between PFAPA and aphthous stomatitis, abdominal pain, or arthralgia. Patients with PFAPA were significantly less likely to experience diarrhoea (p=0.007) and chest pain (p=0.003). Treatment modalities included steroids (45.6%), tonsillectomy (57.9%), colchicine (91.2%), and antibiotics (56.1%).

**Conclusions:**

This study offers important perception into the clinical characteristics, treatment outcomes, and epidemiology of PFAPA syndrome in Palestinian patients. The findings highlight fever and pharyngotonsillitis as predominant symptoms, along with significant associations observed with adenitis. Treatment approaches involving steroids, tonsillectomy, colchicine, and antibiotics were frequently utilised, with notable responses reported.

## INTRODUCTION

PFAPA syndrome, which stands for Periodic Fever, Aphthous Stomatitis, Pharyngitis, and Adenopathy, is an autoinflammatory disorder with an unknown cause. It is marked by episodes of high fever lasting 3–7 days that recur every 2–8 weeks, accompanied by symptoms such as aphthous stomatitis, pharyngitis, and cervical adenitis.^1–3^ Initially described by Marshall et al. in 1987 as an unidentified periodic fever syndrome affecting children under 5 years old, it was later named PFAPA syndrome.^4^

The diagnosis of PFAPA primarily depends on clinical classification criteria.^5^ The diagnostic criteria were first suggested by Marshall et al., indicating that symptoms should begin before the age of 5. Although this is commonly the case, it is now well-acknowledged that PFAPA can also onset later in life, including adulthood.^2^

Currently, PFAPA syndrome is recognised as a distinct clinical entity and is considered the most prevalent autoinflammatory disease. However, there remains some debate regarding the diagnostic criteria. Eurofever proposes a novel set of validated classification criteria for PFAPA with high sensitivity (0.97) and specificity (0.93).^13^

During flare-ups of PFAPA syndrome, laboratory results typically reveal nonspecific abnormalities such as leucocytosis with neutrophilia and elevated levels of erythrocyte sedimentation rate (ESR), C-reactive protein (CRP), serum amyloid A (SAA), and fibrinogen. In some cases, serum IgD may also be increased.^6,7^

The exact cause of PFAPA remains unknown, but it seems to be linked to genetic predisposition involving variations in genes such as MEFV, NLRP, TNFRSF1A, CARD15/NOD2, and MVK. Inflammasomes, which are intracellular proteins that play a role in innate immunity by activating interleukin-1b (IL-1b) and IL-18, are thought to contribute to PFAPA’s pathogenesis. However, studies suggest that no single gene is associated with PFAPA, indicating it may have a multifactorial or polygenic origin, with environmental triggers potentially initiating inflammasome activation and PFAPA flares.^2,8–10^

PFAPA typically follows a benign and self-limiting course. The primary treatment involves low-dose corticosteroids, which quickly alleviate flares but do not prevent future episodes. Tonsillectomy has been successful in some children. Additionally, colchicine, cimetidine, nonsteroidal anti-inflammatory drugs, and interleukin-1 inhibitors have shown effectiveness.^11,12^

The aim of this study is to enhance understanding of PFAPA syndrome in Palestinian paediatric patients, addressing the controversies surrounding its diagnostic criteria and the unknown epidemiology in this region. Specifically, the study seeks to describe the clinical characteristics of PFAPA, including the age of onset, associated signs and symptoms during fever episodes, laboratory findings, treatments, and family history of recurrent fever and tonsillectomy.

## MATERIALS AND METHODS

This observational quantitative study employs a retrospective cohort design to investigate the course of PFAPA syndrome in Palestinian paediatric patients. Data was collected from the medical records of the patients under 18 years of age diagnosed with PFAPA at hospitals and paediatric clinics in the at the Al-Ahli, and Palestinian Red Crescent Society (PRCS) Hospitals, Hebron, West Bank, Palestine, over a five-year period from January 1, 2018, to December 31, 2023. The study population includes children diagnosed with PFAPA during the study period.

Inclusion criteria encompass all children up to 18 years old at the time of diagnosis who satisfy the Eurofever/PRINTO clinical classification criteria for PFAPA, which requires meeting at least seven out of eight criteria: presence of pharyngotonsillitis, episode duration of 3–6 days, cervical lymphadenitis, and periodicity, and absence of diarrhoea, chest pain, skin rash, and arthritis. Patients diagnosed with other causes of fever such as Familial Mediterranean Fever (FMF), other Hereditary Periodic Fever syndromes (HPFs), and cyclic neutropenia, as well as those not meeting the Eurofever/PRINTO criteria were excluded.

Patients with this syndrome often referred to rheumatological clinic, which available in 2 centres at Hebron city (Al-Ahli & PRCS),

## STATISTICAL ANALYSIS

Data entry and analysis were conducted using Statistical Package for Social Sciences (SPSS) version 23 software. Descriptive analysis was employed to summarise the characteristics of the participants, presenting categorical variables as frequencies and percentages, and continuous variables as mean ± standard deviation (SD) or medians and interquartile ranges. To compare groups, the Chi-squared test and independent t-test, or their nonparametric alternatives, were utilised as appropriate. A p-value of less than 0.05 was considered statistically significant. The reliability of the scales used in this study was assessed using Cronbach’s alpha.

## RESULTS

**Figure 1.**
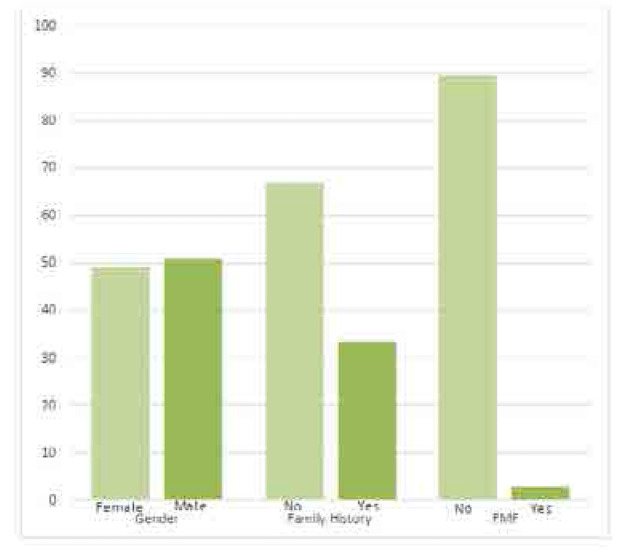
Demographic characteristics of the study participants.

In our study, we admitted 100 patients are diagnosed clinically with PFAPA by the diagnostic Marshall criteria, then classified by Eurofever criteria, and all patients who get 7 points or more were included in our study, a total of 57 patients obeyed the Eurofever / PRINTO criteria and PFAPA diagnosis was confirmed, the remaining 43 patients were managed clinically as PFAPA, but they didn’t meet the criteria, so they were excluded from our study. The results showed that 50% of the participants were females and 50% were males. Of those, 49.1% of females had PFAPA syndrome and 50.9% of males had PFAPA syndrome (**Figure 1**). The mean age of patients who had PFAPA syndrome was 4.17 ± 2.5.

The results revealed that 66.7% (n=38) of the participants had no family history of hereditary periodic fevers, whereas 33.3% (n=19) had family history of hereditary periodic fevers There are no significant differences between participants who had PFAPA syndrome attributed to the family history (p=0.253).

The results revealed that 89.5% (n=51) of the PFAPA patients had no FMF syndrome, whereas 10.5% (n=6) had FMF syndrome, these patients diagnosed with FMF then developed symptoms of PFAPA, all of them underwent genetic testing to confirm FMF diagnosis, 4 patients with heterozygous mutation in E148Q gene, 1 patient with compound heterozygous mutations in E148Q and V726A genes, 1 patient with homozygous mutation in M694V gene, the diagnosis of both diseases made by clinical presentation.

With respect to the clinical features of patients with PFAPA, the attack was recurrent and periodic in most patients 98.2% (n=56), with the mean duration of attack per day was 3.47 ± 1.7. Similarly, the mean interval between attacks per week was 4.56 ± 1.8.

The results showed that 93.0% (n=53) of the PFAPA patients had fever, whereas 7.0% (n=4) did not have fever (P=0.946) (**Table 1**). In addition, all PFAPA patients 100.0% (n=57) had pharyngotonsillitis. The results indicated that pharyngotonsillitis is the most common symptom during febrile episodes (P=0.006). This suggests that PFAPA is related to the likelihood of experiencing pharyngotonsillitis. Similarly, the results showed that there is a significant association between having PFAPA and the presence of adenitis (P=0.001). With respect to aphthous stomatitis, 38.6% (n=22) of PFAPA patients confirmed having aphthous stomatitis and 61.4% (n=35) did not have aphthous stomatitis. The results showed that there is no significant association between having PFAPA and the presence of aphthous stomatitis (P=0.864). In addition, there is no significant association between having PFAPA and the presence of abdominal pain (p=0.199), or arthralgia (p=0.818). The results showed that 3.5% (n=2) of the PFAPA patients had diarrhoea, whereas 96.5% (n=55) did not have diarrhoea. The results indicated that there is an association between having PFAPA and the absence of diarrhoea (P=0.007). All PFAPA patients (100.0%, n=57) reported that they did not have chest pain.

**Table 1.** Characteristics of episodes and symptoms.

	**PFAPA patients (n = 57)**
The mean duration of attack per day	3.47 ± 1.7
The mean interval between attacks per week	4.56 ± 1.8
Recurrent fever	93.0% (n=53)
Pharyngotonsillitis	100.0% (n=57)
Adenitis	75.4% (n=43)
Aphthous stomatitis	38.6% (n=22)
Abdominal pain	52.6% (n=30)
Periodicity	98.2% (n=56)
Diarrhoea	3.5% (n=2)
Chest pain	0% (n=0)
Arthralgia	49.1% (n=28)
Arthritis	0% (n=0)
Rash	0% (n=0)

The results reported that all PFAPA patients (100.0%, n=57) did not experience arthritis and a rash. There is no association between having PFAPA and the presence of arthritis (p=0.152).

**Figure 2.**
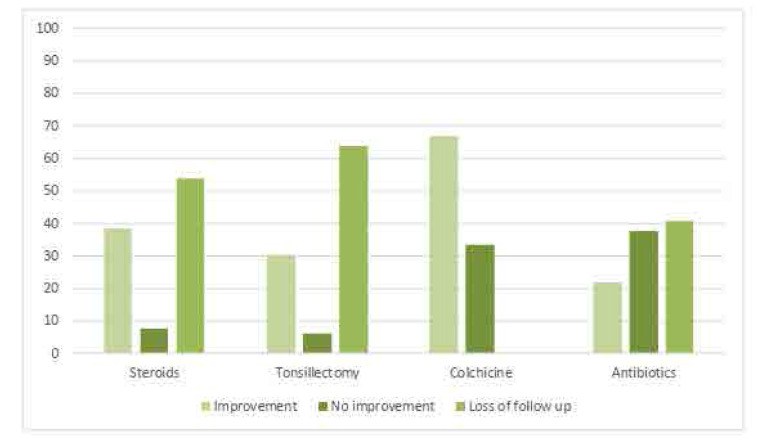
Outcome of treatment.

Regarding treatment (**Figure 2**), most patients tried multiple treatment options, 20 patients were lost to follow up. 45.6% of PFAPA patients (n=26) were prescribed steroids to abort single febrile episodes whereas 54.4% (n=31) did not take steroids. The effect of steroids was documented in 12 patients, 10 of whom reported total improvement of symptoms after a single dose at the starting of each attack, 2 reported no response after steroid, and 14 patients were lost to follow up, patients who was treated with steroids were asked to return for follow up 3–6 months after starting the treatment.

Also, 57.9% (n=33) of PFAPA patients underwent tonsillectomy whereas 42.1% (n=24) did not undergo tonsillectomy. The effect of tonsillectomy was documented in 12 patients, 10 of whom reported total improvement & no attacks after the surgery were noticed, and 2 reported recurrences of attacks after surgery. The findings revealed that most PFAPA patients (91.2%, n=52) did not take colchicine treatment whereas 8.8% (n=5) took colchicine treatment. The effect of colchicine was documented in 3 patients, 2 of whom reported decrease in frequency and duration of symptoms since starting the colchicine, and 1 reported no improvement. Also, more than half of the PFAPA patients (56.1%, n=32) took antibiotics whereas 43.9% (n=25) did not take antibiotics. The effect of antibiotics was documented in 19 patients, 7 of whom reported partial improvement, 12 reported no improvement, and 13 patients were lost to follow up.

## DISCUSSION

Patients who present with recurrent fever, aphthous stomatitis, pharyngotonsillitis, & adenitis are suggested to have PFAPA syndrome, which is a rare periodic fever syndrome first described in the 1980s. Since then, PFAPA syndrome has gained increasing recognition and has been characterised in patient cohorts worldwide.

A 2022 study in western Sweden highlighted the age distribution of PFAPA onset, noting that nearly 90% of affected children first experienced symptoms before age 5, with fewer than 3% presenting symptoms after age 10. Similarly in our study, most patients had their first attack before the age of 5, with mean age of onset 4.17 ± 2.5.

Pharyngitis was the most common symptom during febrile episodes, followed by cervical adenitis and aphthous stomatitis. Approximately 14% of patients exhibited atypical features, with skin rash being the most prevalent.^2^ Our study showed a similar distribution of typical symptoms, whereas the most common atypical symptom was abdominal pain with 53%.

The diagnostic criteria for PFAPA syndrome were originally proposed by Marshall et al.,^4^ emphasising symptom onset before 5 years of age, though recent recognition indicates symptom onset can occur later, even into adulthood. This has prompted the acknowledgment of the need for new, evidence-based criteria, recently proposed but not yet independently validated, aimed at creating homogeneous patient groups in research rather than clinical practice.^4^ Eurofever has proposed a novel, validated set of classification criteria for PFAPA syndrome with high sensitivity (0.97) and specificity (0.93). These criteria include both positive (presence) and negative (absence) clinical variables, resulting in a significantly improved accuracy (0.99). According to Eurofever/PRINTO criteria, a diagnosis of PFAPA requires the presence of at least seven out of eight criteria, including pharyngotonsillitis, episodes lasting 3–6 days, cervical lymphadenitis, and periodicity, while excluding diarrhoea, chest pain, skin rash, and arthritis,^13^ this criterion was used to include or exclude patients in our study.

Incidence data for PFAPA syndrome is limited. Forsvoll et al.^14^ reported an annual incidence of 2.3 per 10,000 children up to 5 years old, based on a study of 34 children at a university hospital in Norway. Renko et al.’s review suggested similar figures, estimating an annual incidence of 2 per 10,000 children < 5 years old in Finland, drawn from a cohort of 133 patients with periodic fevers who underwent tonsillectomy, where at least 34 did not meet modified Marshall criteria due to fever being the sole symptom during episodes.^4^

In our study in the south West Bank, the incidence was calculated to be 1.186 per 10,000 children under 18 years old who were diagnosed and obeyed Eurofever/PRINTO criteria.

A 2014 study emphasised the importance for paediatricians and ENT specialists to consider PFAPA syndrome in cases of recurrent fever attacks before initiating antibiotics. Colchicine treatment was found effective in reducing the frequency of fever episodes.^3^ Two out of three patients who was treated with colchicine reported improvement, whereas seven of nineteen showed improvement on antibiotics.

The best way to treat patients with PFAPA is still a somewhat controversial issue. In this cohort, tonsillectomy was performed in 33 patients. Unfortunately, follow-up data were not available for all patients, but more than 83% of patients with available data after tonsillectomy reported a complete or partial resolution of symptoms after the procedure. This compares with the findings in a recently conducted review that found surgery curative in 92% of patients. Families should be carefully informed of the potential risks and benefits of the procedure, and a shared decision-making approach should be used. Future studies are needed to determine the best treatment regime for PFAPA.

Overall, studies consistently show that PFAPA syndrome predominantly affects young children, with a peak in 1-year-olds, and tends to diminish in older age groups. Symptoms and clinical presentation are similar regardless of whether symptoms begin before or after 5 years of age. Marshall et al.’s initial description in 1987 noted that most patients experienced symptoms before age 5, highlighting the syndrome’s prevalence in younger children despite cases with later onset. The immunological mechanisms underlying PFAPA in relation to the developing immune system of young children remain an intriguing area for further research. Although PFAPA syndrome is well recognised as a clinical entity and is generally regarded as the most common autoinflammatory disease, this study clarified some of clinical presentations and epidemiology of the syndrome in Palestine, there is still some controversy regarding the diagnostic criteria. Moreover, this is the first paper in the West Bank that discusses this rare syndrome, and we encourage further studies and investigations to better understand PFAPA in Palestine. Many of the patients presented with symptoms of PFAPA were misdiagnosed as tonsillitis or FMF, and managed without putting PFAPA as differential, in addition that many patients diagnosed with the syndrome losses the follow up after establishing the effective treatment.

## CONCLUSION

This study provides valuable insights into the clinical features, treatment outcomes, and epidemiology of PFAPA syndrome among Palestinian patients. The findings underscore the prevalence of fever and pharyngotonsillitis as hallmark symptoms, alongside significant associations with adenitis. Treatment strategies such as steroids, tonsillectomy, colchicine, and antibiotics were commonly employed, with notable responses reported. These results contribute to the broader understanding of PFAPA syndrome and emphasise the need for further research to refine diagnostic criteria and enhance management strategies in clinical practice.

## AUTHORS’ CONTRIBUTIONS

RW, RQ, and RAD contributed to design of the study, data analysis, data interpretation, and drafting of the manuscript. ONS and FAN contributed to design of the study, data collection, data entry, and data interpretation. ONS and FAN contributed to design of the study, data interpretation, drafting of the manuscript, and supervision of the work. All authors have read and approved the final manuscript. Each author has participated sufficiently in the work to take public responsibility for the content.

## ETHICS CONSIDERATION

All methods were conducted in accordance with the ethical standards of the declaration of Helsinki. / Accordance with relevant guidelines and regulations. Ethical approval for this study was obtained from the Palestinian Ministry of Health for data collection and also from the Research Ethics Committee — faculty of medicine, Hebron University. The data were collected anonymously without subjects or gender identification. All responses were encoded, used primarily for statistical analysis, and treated confidentially. According to the culture and traditions where the study was conducted, there were no social risks or harm.

## CONSENT FOR PUBLICATION

Not applicable.

## AVAILABILITY OF DATA AND MATERIALS

The dataset used and analysed during the current study is available from the corresponding author upon reasonable request.

## CONFLICT OF INTEREST

All authors declare no conflicts of interest.

## FUNDING

No funding was received for this study.
